# The interplay between translational efficiency, poly(A) tails, microRNAs, and neuronal activation

**DOI:** 10.1261/rna.079046.121

**Published:** 2022-06

**Authors:** Timothy J. Eisen, Jingyi Jessica Li, David P. Bartel

**Affiliations:** 1 Howard Hughes Medical Institute, Cambridge, Massachusetts 02142, USA; 2 Whitehead Institute for Biomedical Research, Cambridge, Massachusetts 02142, USA; 3 Department of Statistics, Department of Biostatistics, Department of Computational Medicine, and Department of Human Genetics, University of California, Los Angeles, California 90095, USA

**Keywords:** poly(A) tails, microRNAs, neurons, translational efficiency, PAL-seq, ribosome profiling

## Abstract

Neurons provide a rich setting for studying post-transcriptional control. Here, we investigate the landscape of translational control in neurons and search for mRNA features that explain differences in translational efficiency (TE), considering the interplay between TE, mRNA poly(A)-tail lengths, microRNAs, and neuronal activation. In neurons and brain tissues, TE correlates with tail length, and a few dozen mRNAs appear to undergo cytoplasmic polyadenylation upon light or chemical stimulation. However, the correlation between TE and tail length is modest, explaining <5% of TE variance, and even this modest relationship diminishes when accounting for other mRNA features. Thus, tail length appears to affect TE only minimally. Accordingly, miRNAs, which accelerate deadenylation of their mRNA targets, primarily influence target mRNA levels, with no detectable effect on either steady-state tail lengths or TE. Larger correlates with TE include codon composition and predicted mRNA folding energy. When combined in a model, the identified correlates explain 38%–45% of TE variance. These results provide a framework for considering the relative impact of factors that contribute to translational control in neurons. They indicate that when examined in bulk, translational control in neurons largely resembles that of other types of post-embryonic cells. Thus, detection of more specialized control might require analyses that can distinguish translation occurring in neuronal processes from that occurring in cell bodies.

## INTRODUCTION

**FIGURE 1.**
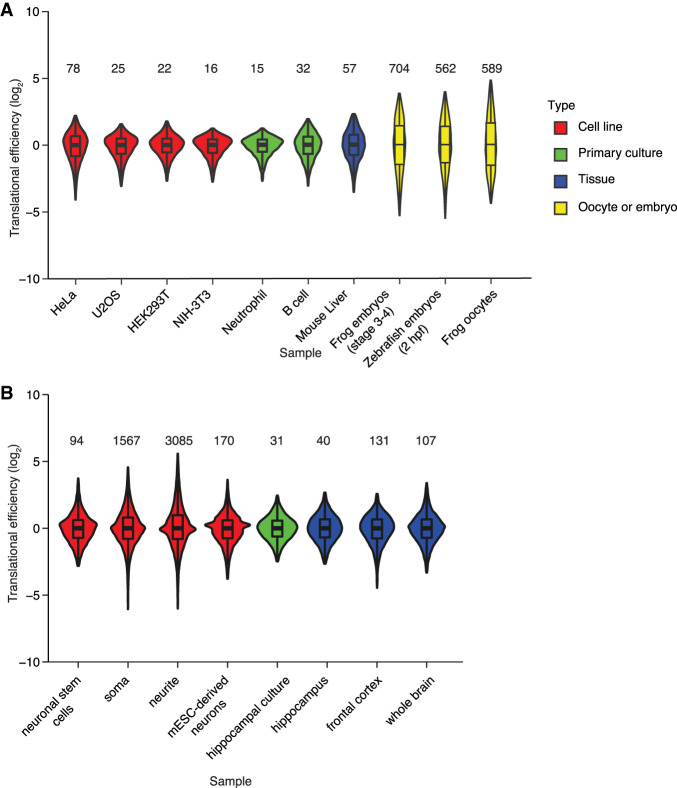
Larger ranges in TE observed in oocytes and early embryos. (*A*) Ranges in TE values acquired from a variety of sample types (key) by the same laboratory. Each distribution is represented as a violin plot with box-and-whisker overlay, after median centering and removing outliers falling beyond the 1–99th percentiles (line, median; box, 25th–75th percentiles; whiskers, 1.5 times the interquartile range). The 1–99 percentile range is printed *above* the distribution. Values from HeLa cells (*n* = 6918), HEK293T cells (7610), mouse liver (3716), NIH 3T3 cells (7446), frog embryos (6105), and zebrafish embryos (4161) are from Subtelny et al. (2014), those from U2OS cells (7525) and B cells (5593) are from Eichhorn et al. (2014), those from neutrophils (6551) are from Guo et al. (2010), and those from frog oocytes (11,371) are from Xiang and Bartel (2021). (*B*) Ranges in TE values acquired from neuronal tissues and neuronal cultures by different laboratories. Distributions are represented as in *A*. Values from adult neural stem cells (*n* = 12,623) are from Liu et al. (2018), those from soma (22,298) and neurites (22,398) of neurons derived from mouse embryonic stem cells (mESCs) are from Zappulo et al. (2017), those from mESC-derived neurons (22,053) are from Gonatopoulos-Pournatzis et al. (2020), those from primary hippocampal culture (10,333) and hippocampal tissue (10,333) are from Cho et al. (2015), those from frontal cortex (16,158) are from Das Sharma et al. (2019), and those from whole brain (15,766) are from Hornstein et al. (2016).

In yeast and mammalian cell lines often used for high-throughput molecular and biochemical studies, regulation of mRNA production and turnover account for much of the variance in the protein produced from different genes (Schwanhausser et al. 2011; Li et al. 2014; Weinberg et al. 2016). Accordingly, global techniques, such as RNA sequencing (RNA-seq), ribosome profiling, and mass spectrometry show that the relationship between mRNA levels and protein output can be a close one (Schwanhausser et al. 2011; Li et al. 2014). For example, ribosome-footprint profiling from our laboratory indicates that mRNAs in NIH 3T3 cells (a mouse fibroblast line) have a narrow range of translational efficiency (TE) values (calculated as the number reads for ribosome-protected fragments [RPFs] divided by the number of RNA-seq reads), with the 1–99 percentile range spanning only 16-fold (Fig. 1A; Eichhorn et al. 2014). Likewise, relatively narrow TE ranges (<80-fold) are observed in HeLa, U2OS, and HEK293T cells, as well as mouse tissues and primary cultures, such as liver, neutrophils, and B cells (Fig. 1A; Eichhorn et al. 2014; Subtelny et al. 2014). In contrast, in metazoan oocytes and early embryos, where transcription is silenced and mRNAs are quite stable, protein production is primarily regulated at the level of differential translation. Accordingly, in oocytes and early embryos, mRNAs have a broader range of TE values, with the 1–99 percentile range spanning >560-fold (Fig. 1A; Subtelny et al. 2014; Xiang and Bartel 2021).

The brain might be the post-embryonic tissue with the greatest amount of translational control (Costa-Mattioli et al. 2009; Buffington et al. 2014; Kapur et al. 2017; Holt et al. 2019). In the brain, the signals of learning and memory are integrated throughout rapidly responding networks that alter the proteome in response to stimuli. Decades of studying these expression changes have motivated exploration of the role of translational control at a global scale. However, ribosome profiling in neurons and brain tissues has not consistently indicated a broad range of TE values (Fig. 1B). At one extreme, a data set from neurites derived from embryonic-stem cells has a 1–99 percentile range spanning 3100-fold, whereas at the other extreme, a data set from the hippocampus has a 1–99 percentile range of only 40-fold (Fig. 1B; Cho et al. 2015; Hornstein et al. 2016; Blair et al. 2017; Zappulo et al. 2017; Liu et al. 2018; Umegaki et al. 2018; Das Sharma et al. 2019; Rodriguez et al. 2019; Biever et al. 2020; Simbriger et al. 2020). These differences observed by different labs in different studies might be biological, reflecting true differences in ranges for different cell types or different culture conditions. However, some differences might be attributable to different experimental protocols. For example, some methods for mRNA purification can impose biases that artificially broaden the apparent range in TE values (Weinberg et al. 2016).

In oocytes and early embryos, the large differences in TEs are best understood and largely attributable to differences in poly(A)-tail lengths, with longer tails causing greater translation. In these settings, TE correlates with mean poly(A)-tail length with Spearman coefficients (*R*_S_ values) ranging from 0.61–0.77 (Subtelny et al. 2014; Xiang and Bartel 2021). Tail-length changes can be specified by elements residing in the mRNAs, typically in 3′-UTRs, which are then bound by regulatory factors. For example, CPEB binds to the cytoplasmic polyadenylation element (CPE) and recruits the Gld2 poly(A) polymerase to the mRNA, causing extension of the mRNA tail (Richter 1999; McFleder et al. 2017). Likewise, many repressors bind to 3′-UTR elements and recruit deadenylase complexes that shorten the tail (Kim and Richter 2006; McFleder et al. 2017). For example, microRNAs (miRNAs) pair to sites in mRNAs to direct their deadenylation (Bartel 2018). MicroRNAs accomplish deadenylation through their association with an Argonaute (AGO) protein, which recruits the TNRC6 scaffolding protein, which in turn recruits deadenylase complexes—primarily the CCR4–NOT complex (Jonas and Izaurralde 2015).

When considering the importance of poly(A)-tail length for specifying TE in oocytes and early embryos, it is perhaps surprising that coupling between tail length and TE diminishes at gastrulation, and that no global relationship between tail length and TE has been observed in the post-embryonic tissues and cell lines that have been examined (Subtelny et al. 2014). In these post-embryonic contexts, mRNAs with shorter tails are translated just as efficiently as those with longer tails (Subtelny et al. 2014). Here, tail shortening reduces mRNA stability rather than TE, and short-tailed isoforms that had previously undergone accelerated deadenylation are degraded more rapidly (Eisen et al. 2020b). This developmental transition in regulatory regimes, in which the consequences of tail shortening change from reduced translation to reduced stability, explains a concurrent shift in the molecular consequences of miRNAs. In early embryos, miRNA-mediated tail shortening causes reduced translation with no decrease in the amount of the mRNA, whereas later in development miRNA-mediated tail shortening primarily reduces mRNA levels with relatively little change in TE (Guo et al. 2010; Eichhorn et al. 2014; Subtelny et al. 2014; Eisen et al. 2020a). Thus, in all post-embryonic cells and tissues examined using global analyses, most miRNA-mediated repression is attributable to destabilization of target mRNAs, and any additional miRNA-mediated reductions in TE that are sometimes detected cannot be attributed to tail shortening because tail length and TE are not coupled in these cells.

The abundance of translational control mechanisms described in brain tissues and neuronal culture, and some reports of a broader range of TE values in these contexts, raise the possibility that the regulatory regime operating in neurons might differ from that of other post-embryonic cells that have been examined and might instead resemble the regulatory regime operating in oocytes and early embryos. Indeed, a CPE is found in a highly expressed, activity-induced neuronal mRNA, *Camk2a*, and appears to cause poly(A)-tail lengthening of this mRNA in response to stimulation, both in cultured neurons and brain tissues (Wu et al. 1998; Dziembowska et al. 2012). Moreover, other mRNAs might also be substrates for cytoplasmic polyadenylation in neurons, as suggested from results of experiments using differential thermal elution from poly(U)-sepharose columns, which identify tens to hundreds of additional candidates, many of which contain the canonical CPE motif (Du and Richter 2005; Udagawa et al. 2012). These reports imply that TE might be coupled to tail length in neurons, as it is in oocytes and early embryos. Nonetheless, the overall impact of tail lengths and cytoplasmic polyadenylation in neurons is unclear, with fundamental questions remaining regarding the number of molecules affected, the extent of tail lengthening, the effect of the phenomenon on translation, and whether it is cell-wide or distal-dendrite specific in its subcellular localization (Wu et al. 1998; Du and Richter 2005). Moreover, in neurons, miRNAs cause destabilization of their mRNA targets (Tan et al. 2013), suggesting a regulatory regime resembling that of other post-embryonic cells, although the prospect of a more consequential effect of miRNAs on TE in neurons has not been investigated using global measurements. Here, we combined transcriptome-wide measurements of TE and poly(A)-tail length in mouse primary-culture neurons and brain tissues to investigate the mRNA features underlying TE in these contexts and the potential effects of miRNAs and neuronal activation.

## RESULTS

### Stimulus-induced changes in TE are modest

**FIGURE 2.**
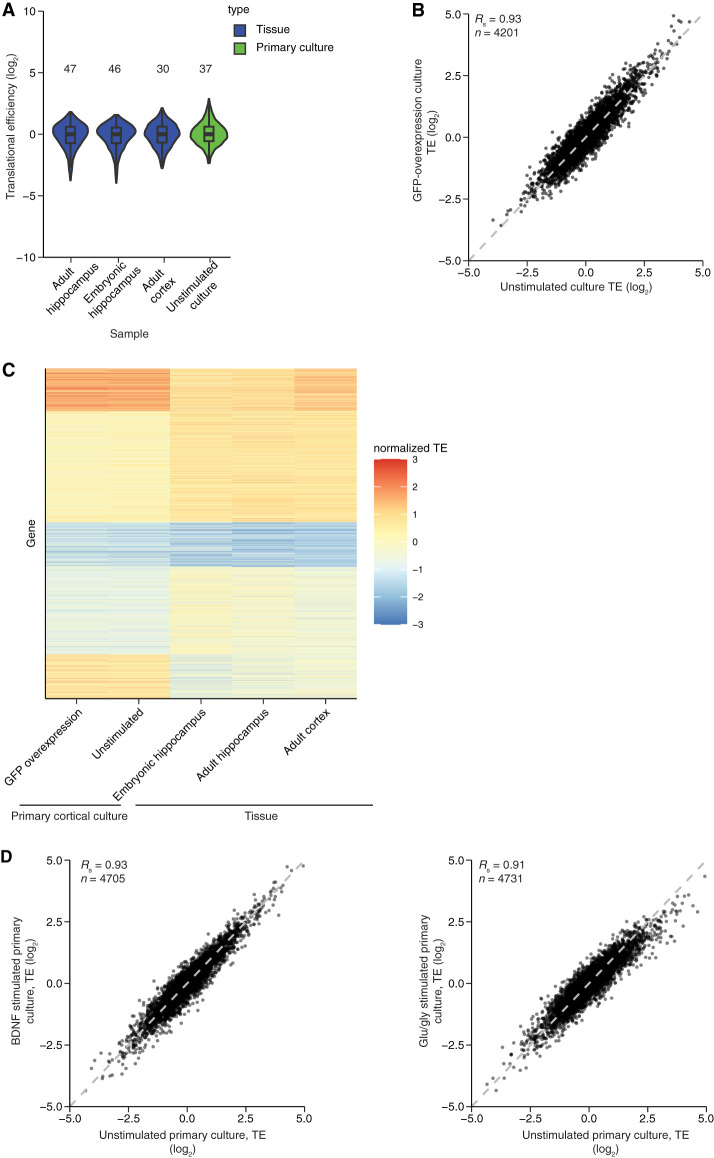
TE values from neuronal tissues and cultures with and without stimulation. (*A*) Ranges in TE values acquired for unstimulated neuronal samples in this study. The 1–99 percentile range is printed *above* the distribution. Values are from adult hippocampus (6342), embryonic hippocampus (7443), adult cortex (5019), and unstimulated cortical culture (*n* = 5129). Otherwise, this panel is as in Figure 1A. (*B*) Reproducibility of TE measurements. Shown is the relationship between the TE values for mRNAs from an unstimulated primary cortical culture and those from an analogous culture in which GFP was expressed from a lentiviral vector. (*C*) Comparison of TE values obtained from different neuronal samples. The heatmap indicates TE values obtained from mRNAs that passed the expression cutoffs in all five of the indicated samples (*n* = 3347). For each sample, values were *z*-score normalized and then clustered using *k*-means. The gene index is plotted as a function of sample and colored by normalized TE (key). (*D*) Modest effect of stimulation on TE values. Cortical cultures were stimulated with either BDNF (*left*) or glu/gly (*right*), and TE values were compared with those observed without stimulation (*n* = 4705 and 4731, respectively).

To begin to explore the extent of translational control in the brain, we performed ribosome-footprint profiling and matched fragmented RNA-seq from mouse embryonic and adult hippocampus and adult cortex. As expected, the RPFs from these samples mapped preferentially to coding sequences (CDSs) (Supplemental Fig. S1A) and had 3-nt periodicity across the CDSs (Supplemental Fig. S1B–D; Ingolia et al. 2009; Guo et al. 2010). After normalizing to the matched RNA-seq to infer TE values for mRNAs from different genes, the observed 1–99 percentile ranges in values spanned 30- to 47-fold (Fig. 2A). Although broader than the distributions observed in some post-embryonic cells and tissues, these distributions of TE values resembled those with more modest ranges in TE values observed in previous neuronal data sets (Fig. 1A,B). Furthermore, TE values for mRNAs from individual genes correlated with those previously reported, despite some variability in values observed among data sets from different studies (Supplemental Fig. S2).

The brain tissues that we and others dissected and profiled are composed of many cells of distinct lineages, including many nonneuronal cells. To obtain a population enriched for neurons, we cultured primary neurons from the cortex of early postnatal animals for 14 d in vitro (DIV14) in the presence of cytosine arabinoside (ara-C, a potent poison of dividing cells). A range of TE values resembling that of the neuronal tissues was observed (Fig. 2A, 37-fold 1–99 percental range), which implied that nonneuronal cells of the tissues were not dampening an otherwise extended range in TE values. The values observed in DIV14 cortical neurons were reproducible across multiple samples (Fig. 2B) but substantially differed from those of brain tissues (Fig. 2C), presumably due to the effects of culture outside the animal, as well as the elimination of other cell types, which not only removed their contribution to the profiles but also presumably affected the physiology of the remaining neurons (Banker and Goslin 1988). Indeed, the TE values of the adult cortex resembled those of other brain tissues more closely than they resembled those of DIV14 cortical cultures (Fig. 2C).

Although modest, the range in TE values observed in neurons raised the prospect that TE changes might help mediate the response to neuronal stimulation. To investigate this at a global level, we examined the changes in TE after stimulating DIV14 cortical neurons with either BDNF (Ghosh et al. 1994) or glutamate and glycine (glu/gly) to mimic long-term potentiation (LTP) induction (Lu et al. 2001). As expected for successful stimulation, immediate-early genes (IEGs) were strongly up-regulated. These up-regulated IEGs included *Npas4* (18- and 14-fold increase for BDNF and glu/gly stimualtion, respectively), *Arc* (12- and 3.9-fold increase for BDNF and glu/gly stimulation, respectively), and *Fos* (10- and 5.9-fold increase for BDNF and glu/gly stimulation, respectively). After 30 min of either stimulation, TE changes were dwarfed by the constitutive range in TE values observed for mRNAs from different genes (Fig. 2D). These relatively modest changes observed after stimulation indicated that although translational repression sets the stage for neuronal stimulation (Scheetz et al. 2000), differential translation of key activity-regulated mRNAs does not precede neurophysiological changes in a manner that can be detected using our global approach. This result was consistent with the report that, although substantial changes to mRNA levels could be detected in the hippocampus 4 h after fear conditioning, very few TE changes were observed 30 min after conditioning (Cho et al. 2015). The previous study does identify one translationally regulated mRNA, *Nrsn1*, which is reported to be 1.4-fold down-regulated upon stimulation. Although not expressed highly enough for analysis in our primary-culture experiment, we note that even without added stimulation of adult hippocampus, this mRNA was translated less efficiently than 97% of the mRNAs examined. Thus, further investigation of such a function for differential translational control will require either more sensitive or more directed experimental approaches.

### MicroRNAs predominantly act to decrease target mRNA abundance in neurons

When considering the possible mechanisms that might help impart the translational regulation observed in neurons, we wondered about miRNAs. Although miRNA-mediated repression is predominantly attributed to mRNA destabilization in all post-embryonic contexts examined, a minor component of the repression attributable to translational repression is sometimes detected. This translational repression component might be more impactful in neurons, since neurons are reported to use cytoplasmic polyadenylation to target specific mRNAs for increased translation (Wu et al. 1998; Du and Richter 2005; Dziembowska et al. 2012; Udagawa et al. 2012), which implies a post-transcriptional regulatory regime resembling that of early embryos in the sense that translation is sensitive to poly(A)-tail length. In such a regime, coupling between tail length and TE would enable a greater impact of miRNAs on TE, as it does in early embryos. Speaking against this possibility, however, global analyses of the effects of miRNAs on mRNA levels after perturbing neuronal miRNAs, such as miR-128 in the striatum (Tan et al. 2013) or miR-183/96/182 in the retina (Krol et al. 2010), show that miRNAs act to reduce mRNA levels in neurons, implying an important difference between the regulatory regimes of neurons and early embryos with respect to the decay of short-tailed mRNAs. Nonetheless, the global effects of miRNAs on TE had not been reported in neurons, leaving open the possibility that the effects on TE might be greater than those on mRNA levels in these cells.

**FIGURE 3.**
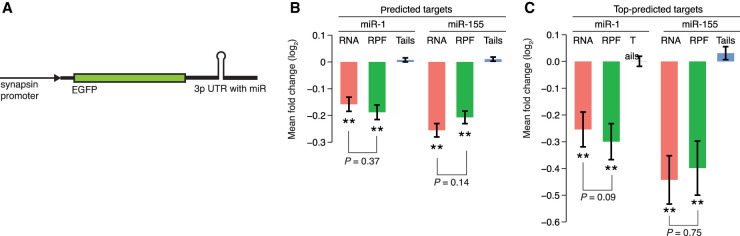
Negligible effect of miRNAs on TE in cultured neurons from mouse cortex. (*A*) Schematic of miRNA-expression constructs. Lentiviral vectors expressed *GFP* mRNAs with 3′-UTRs that had pri-miRNA segments suitable for expressing either miR-1 or miR-155. After packaging, purified virus was used to transduce primary-culture neurons at DIV5. Neurons were harvested for analysis at DIV14. (*B*) Impact of miR-1 (*left*) and miR-155 (*right*) on mRNA levels, ribosome-protected fragments, and mean tail lengths for predicted targets (*n* = 270 and 203 for miR-1 and miR-155, respectively). Values were normalized to those of mRNAs with no site to the transduced miRNA (*n* = 745 and 1017 for no-site cohorts for miR-1 and miR-155, respectively). Significant changes are indicated with asterisks *below* the bar ([*] *P* < 0.05; [**] *P* < 0.001, one-tailed *t*-test). *P* values comparing the RNA and RPF values indicate the results of an unpaired *t*-test. (*C*) Analysis as in *B*, but considering only top-predicted targets (*n* = 39 and 17 for miR-1 and miR-155, respectively).

To investigate this possibility, we used lentivirus to express miRNAs in neuronal cultures and examined the effects of ectopically expressed miRNAs on both mRNA levels and TE values. In these experiments, we transduced DIV5 neurons with a lentivirus expressing either miR-1 or miR-155 from the 3′-UTR of *GFP* (Fig. 3A) and harvested at DIV14 to examine the cumulative effects of miRNAs after they had reached steady-state expression levels. Almost all neurons were fluorescent upon infection, which demonstrated widespread infection and transgene expression (Supplemental Fig. S3A). The cultures also accumulated the exogenous miRNAs (Supplemental Fig. S3B), although the level of neither miRNA reached that of miR-124, a highly expressed endogenous miRNA in neurons, which indicated that the exogenous miRNAs were not expressed at supraphysiological levels. Using RNA-seq to examine the effects on mRNA levels showed that, as observed in the striatum and retina (Krol et al. 2010; Tan et al. 2013), levels of predicted miRNA targets were reduced in our cortical cultures (Supplemental Fig. S3C,D). Moreover, the effects on predicted targets followed the expected hierarchy of site-type efficacy diagnostic of miRNA-mediated repression (Supplemental Fig. S3C,D; Bartel 2009).

Despite the clear signal for miRNA-mediated mRNA destabilization detected by RNA-seq, our ribosome-profiling results provided no evidence for miRNA-mediated translational repression in these primary neuronal cultures. When considering the predicted targets of either miR-1 or miR-155, the changes to RPF levels were not significantly greater than the changes to mRNA levels, which indicated that the reduced numbers of ribosomes on target CDS regions were fully attributable to changes in mRNA abundance (Fig. 3B). The same was observed when considering only the top-predicted targets, defined as mRNAs repressed by either miR-1 or miR-155 in ribosome profiling data from a separate study (Eichhorn et al. 2014), despite the increased repression of these targets (Fig. 3C). Considering only mRNAs enriched in the synaptic neuropil (Cajigas et al. 2012) did not change this result (Supplemental Fig. S3E,F).

In addition to examining effects of miRNAs on mRNA abundance and TE, we examined the effects of miRNAs on steady-state tail lengths, using sequencing-based poly(A)-tail profiling methods (Chang et al. 2014; Subtelny et al. 2014; Eisen et al. 2020b). Consistent with the lack of evidence for miRNA-mediated translational repression, we observed no miRNA-dependent tendency for tails of predicted targets to decrease (Fig. 3B,C). Thus, even if tail shortening reduces TE in neurons, miRNAs could not exploit this relationship to repress steady-state translation in neurons, because in neurons, in contrast to early embryos, miRNAs do not reduce the steady-state tail lengths of their targets. Nearly imperceptible changes to steady-state tail lengths are also observed when miRNAs are expressed in NIH 3T3 cells (Eisen et al. 2020a). Follow-up presteady-state analyses attribute this phenomenon to a two-pronged repressive mechanism, in which miRNAs not only accelerate deadenylation of their target mRNAs but also accelerate degradation of short-tailed target-mRNA isoforms. The accelerated degradation of short-tailed isoforms prevents a buildup of these isoforms, causing nearly equivalent distributions of steady-state tail lengths in the presence or absence of the miRNA (Eisen et al. 2020a). Our finding that neither miR-1 nor miR-155 altered steady-state tail lengths of their respective target mRNAs in DIV14 cortical cultures suggested that this two-pronged mRNA-destabilization mechanism extends to neurons.

In sum, the molecular consequences of miRNA-mediated repression in neurons, with respect to changes (or lack thereof) in target mRNA levels, TE, and steady-state tail lengths, are indistinguishable from those observed in other post-embryonic contexts. Thus, the action of miRNAs and the range of TE values paint a landscape of translational control in neurons that does not fundamentally differ from that of other post-embryonic contexts.

### TE and poly(A)-tail length are modestly coupled in neurons

**FIGURE 4.**
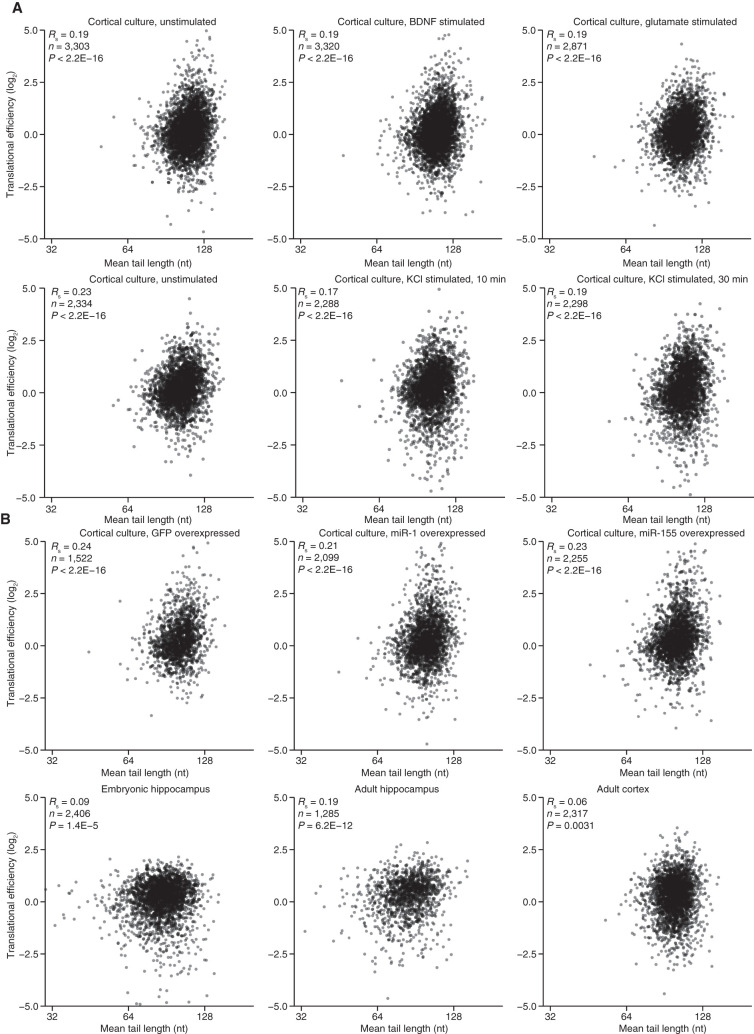
A modest relationship between tail length and TE in primary cortical cultures and tissues. (*A*) Relationship between TE and mean tail length for mRNAs from each gene that passed expression cutoffs for both measurements. Plotted are results from nine primary cortical cultures examined in this study. (*B*) Relationship between TE and mean tail length for tissue samples; otherwise as in *A*.

Although miRNAs do not exploit coupling between tail length and TE by using changes in tail length to alter TE values in neurons, other regulatory phenomena might. If they did, then we would expect to see a strong relationship between tail length and TE in neurons, as is observed in oocytes and early embryos (Subtelny et al. 2014; Eichhorn et al. 2016; Lim et al. 2016; Xiang and Bartel 2021). Therefore, to explore whether this regulatory strategy might explain a substantial fraction of the TE variability observed in neurons, we acquired tail-length profiling data from the three brain tissues examined earlier, and analyzed the relationship between TE and tail length in each of the brain-tissue or cultured-neuron samples for which we had tail-length and TE data (including three samples from cortical cultures examined in our analysis of miRNA effects and six samples from stimulated and control cortical cultures, described later). For mRNAs from the nine primary cortical cultures, a modest relationship between tail lengths and translational efficiencies was repeatedly observed (Fig. 4A, R_s_ = 0.17–0.24, *P* < 10^−16^ for each of the nine samples), indicating that mean poly(A)-tail length explained a small yet detectable amount of the variance in TE values. Weaker correlations were typically observed for mRNAs from the tissue samples (Fig. 4B, R_s_ = 0.06–0.19, *P* ≤ 0.003), perhaps due to the heterogenous nature of these samples.

The coupling between tail length and TE that exists specifically in neuronal cells (albeit with modest effects) might result from features of the neuronal cytoplasm that allow this coupling, such as a regime of limiting cytoplasmic poly(A) binding protein (PABPC) activity (Xiang and Bartel 2021). Alternatively, mRNAs expressed in neurons might contain sequences that enable tail length to influence TE. In the latter scenario, considering only transcripts that are exclusively expressed in neurons might enhance the relationship observed between tail length and translational efficiency. To investigate this possibility, we computationally sorted the mRNAs based on their preferential expression in neurons (Zhang et al. 2014) and evaluated the correlations between tail length and TE for subsets of neuronally enriched mRNAs. In most of the samples, these correlations did not increase more than expected by chance, suggesting that neuronal-biased mRNAs exhibit no more coupling than other mRNAs (Supplemental Fig. S4A). Subsetting the data based on a study that determined mRNAs highly enriched in the hippocampal synaptic neuropil, a region enriched for dendrites, axons and glia, but depleted in neuronal somata (Cajigas et al. 2012), did not significantly improve the correlations (Supplemental Fig. S4B).

### Light stimulation induces tail-length changes in the visual cortex

The weak relationship between tail length and TE observed for mRNAs of brain tissues and cultured neurons raised questions regarding the extent to which neurons use targeted cytoplasmic polyadenylation to modulate gene expression. To begin to address these questions, we monitored changes in tail lengths of thousands of different mRNAs upon visual stimulus of the primary visual cortex. This paradigm is frequently used as a controlled physiological stimulus, as it generates rapid and widespread alterations to large portions of the visual cortex (Majdan and Shatz 2006; Mardinly et al. 2016). In our study, we used tissue from an experiment investigating the diversity of cellular responses to stimulation using single-cell RNA-seq (Hrvatin et al. 2018). The experiment collected visual-cortex tissue from eight mice between 6 and 7 wk old, which had been housed in the dark for 1 wk prior to the dissection. Four of these mice were exposed to light for 1 h prior to dissection, whereas the other four remained in the dark. For each dissected sample, a portion was used for single-cell RNA-seq (Hrvatin et al. 2018), and another portion was used to prepare libraries for tail-length profiling.

Addition of tail-length standards of known length to these libraries enabled quantification of recovery and assessment of tail-length accuracy for RNAs with different tail lengths. Modest depletion of long-tail standards was observed for tail lengths ≳160 nt, with some libraries exhibiting worse depletion than others (Supplemental Fig. S5A). Despite this depletion, tail lengths were called accurately (Supplemental Fig. S5B), and biological replicates showed reasonable agreement between the eight mice (*R*_S_ ≥ 0.65, for all pairwise comparisons of samples without stimulation [*n* = 4552], Supplemental Figure S5C; *R*_S_ ≥ 0.66 for all pairwise comparisons of samples with stimulation [*n* = 4635]; *R*_S_ ≥ 0.59 for pairwise comparison of all eight samples [*n* = 4321]). Furthermore, the depletion of the long-tail standards did not correlate with the stimulation treatment (*P* = 0.841, ANOVA).

**FIGURE 5.**
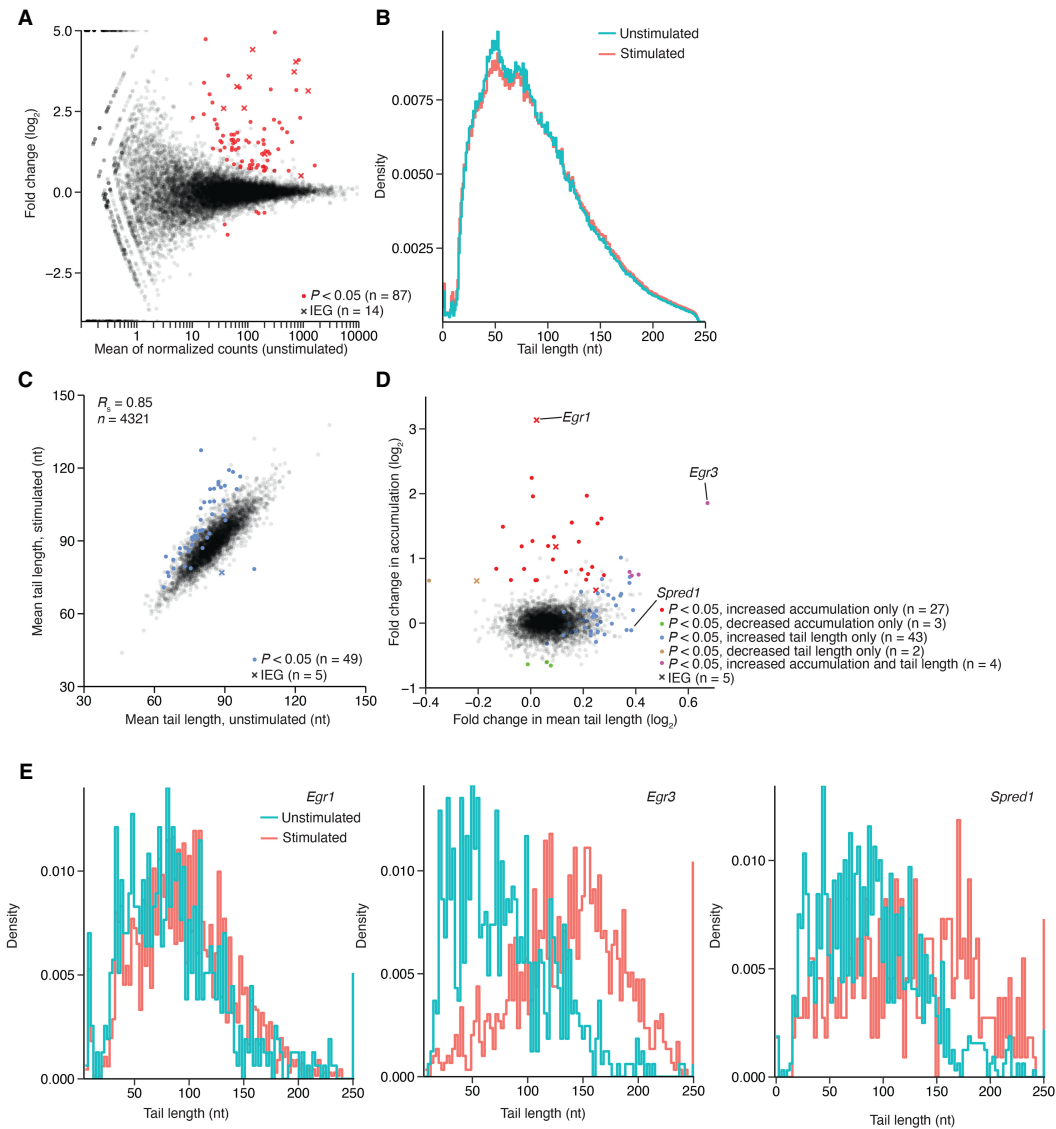
Changes in poly(A)-tail lengths observed in the visual cortex after light stimulation. (*A*) Changes in gene expression observed between the light-stimulated and unstimulated cohorts, as measured using PAL-seq tags. Values for genes with significant changes in expression, as determined by DESeq (Anders and Huber 2010), are in red (adjusted *P* < 0.05, negative binomial test, *n* = 4 mice per cohort), using an “x” to indicate values for an annotated IEG (Tullai et al. 2007). (*B*) The effect of stimulation on the distribution of tail lengths for all tags mapping to annotated genes (*n* = 8,797,239 and 6,236,196 tags for unstimulated and stimulated cohorts, respectively). (*C*) The effect of stimulation on mean tail lengths of mRNAs from each gene. For mRNAs of each gene that passed the expression cutoff, mean tail length in the stimulated cohorts was compared to that in the unstimulated cohorts, highlighting in blue the values that differed significantly in the two cohorts (*P* < 0.05, *t*-test, *n* = 4 values in each cohort). (*D*) Comparison of stimulation-induced change in expression with stimulation-induced change in tail length. Significance testing is as in *A* and *C* (key). (*E*) Histograms showing tail-length distributions for mRNAs of genes labeled in *D*, plotted with 2 nt bins.

Poly(A)-tag abundance indicated relative expression levels and gene-expression changes in the visual cortex upon light stimulation (Fig. 5A). These analyses yielded results consistent with those observed previously (Hrvatin et al. 2018). For example, among the significant changes in mRNA accumulation following stimulation, most changes were in the direction of increased expression, and many were for annotated IEGs (Fig. 5A). *Fosb* (21.3-fold), *Nr4a1* (16.4-fold), and *Fos* (13.2-fold) were the three IEGs with the largest increases in expression, and these increases, along with others from known IEGs (*Egr1*, 8.79-fold; *Npas4*, 6.04-fold; and *Jund*, 1.42-fold) and analyses of single-cell data (Hrvatin et al. 2018), confirmed that stimulation had occurred. When considering the tail lengths for all mRNAs from each sample, stimulation did not change either the shape of the distribution (Fig. 5B) or the mean tail length (*P* = 0.49, ANOVA), and when considering the mean length from mRNAs from each gene, the stimulated samples were not consistently different than the unstimulated samples (Supplemental Fig. S6A). Despite the modest global changes, tail lengths of some mRNAs significantly changed upon stimulation, mostly in the direction of longer tails (Fig. 5C). For one mRNA (*Egr3*), mean tail length increased 47.5 ± 4.5 nt across the four stimulated replicates, and for the other 48 mRNAs with the most statistically significant tail-length increases, mean tail increases ranged from 3.3–27.5 nt.

Stimulation might increase the tail length of mRNAs from an individual gene through several mechanisms. (1) It might increase transcription of the gene (Fig. 5A; Spiegel et al. 2014), in which case, the tails of these nascent molecules, which are generally longer than those of older mRNAs (Eisen et al. 2020b), might not have reached their steady-state length when the tissue was harvested 1 h after stimulation, causing an increase in mean tail length. (2) Stimulation might cause short-tailed isoforms to decay more rapidly without enhancing deadenylation of the longer isoforms, which would increase the mean tail length. (3) Stimulation might induce cytoplasmic polyadenylation (Wu et al. 1998). Each of these potential mechanisms makes a prediction regarding changes in mRNA levels that might accompany changes in tail length. In the first scenario, a tail-length increase would generally be accompanied by an increase in mRNA, whereas in the second scenario, it would generally be accompanied by a decrease in mRNA, and in the third, it would be accompanied by either little change in the mRNA (for mRNAs with slow deadenylation rates and long lifetimes) or an increase in the mRNA (for mRNAs that would have otherwise undergone extensive deadenylation and decay during the span of 1 h).

To investigate these relationships, we compared changes in mRNA accumulation with the changes in mean tail length (Fig. 5D). Four mRNAs (e.g., *Egr3*) had significant increases in both accumulation and mean tail length. Of the mRNAs with significantly increased accumulation, including the 27 mRNAs with no significant change in mean tail length, half (16/31) underwent increased transcription in at least one cell type, as indicated by single-nuclei sequencing from the same samples (Supplemental Fig. S6B; Hrvatin et al. 2018), and most (26/31) had tail-length changes exceeding the median log_2_ fold-change of all mRNAs, which was 0.134. These results supported the idea that tail lengths of some mRNAs increase through the first mechanism in which a burst of nascent transcripts causes a transient increase in tail length. Nonetheless, a few of the strongly induced mRNAs (e.g., *Egr1*) had no detectable tail-length change, presumably because rapid deadenylation had already occurred. Among mRNAs with increased tail lengths, none had either significantly reduced accumulation (Fig. 5D) or a detectable decrease in short-tailed mRNA abundance (Supplemental Fig. S6C), which indicated a lack of support for the second mechanism. Instead, the remaining 43 mRNAs with significantly increased tail lengths (e.g., *Spred1*) had no significant change in accumulation, which was consistent with the third mechanism in which these tails were extended through cytoplasmic polyadenylation (although a more complicated combination of the first and second mechanisms, which in principle might allow tail-length changes without accumulation changes, could not be excluded). Investigation of the individual tail-length distributions of the exemplar mRNAs supported these observations (Fig. 5E). Although some mRNAs with extended tails had a CPE motif, as defined in mammalian oocytes and mouse striatum (Charlesworth et al. 2013; Reyes and Ross 2016; Parras et al. 2018), the presence or absence of a CPE was not significantly associated with tail-length increases (Supplemental Fig. S6D–F), suggesting that the determinants of cytoplasmic polyadenylation in mammalian neurons differ from those identified in frog oocytes.

### Stimulation induces tail-length changes in primary-culture neurons

**FIGURE 6.**
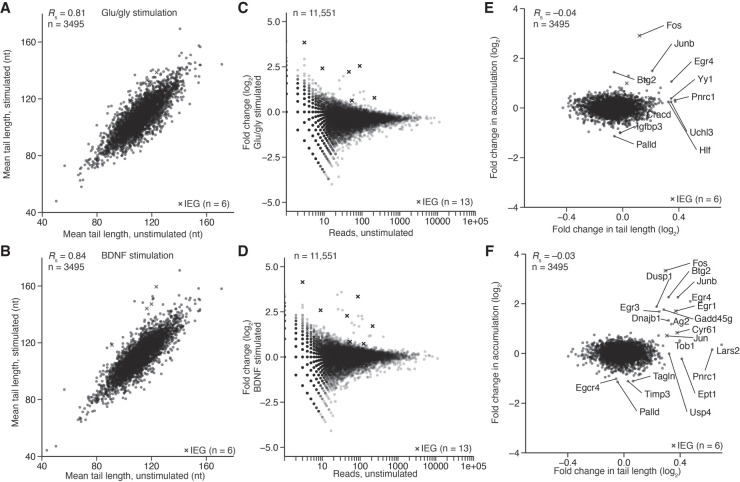
Tail length and expression changes observed during cortical-culture stimulation. (*A*) The effect of glu/gly stimulation on mean tail lengths. For mRNAs of each gene that passed the 50-tag expression cutoff, mean tail length in the stimulated sample was compared to that in the untreated control. Values for IEGs are indicated with an “x.” (*B*) The effect of BDNF stimulation on mean tail lengths; otherwise, as in *A*. (*C*) The effect of glu/gly stimulation on gene expression, as measured by PAL-seq tags. For mRNA of each gene, the fold-change in tags observed upon stimulation is plotted. Otherwise as in *A*. (*D*) The effect of BDNF stimulation on gene expression; otherwise as in *C*. (*E*) Comparison of change in expression with change in tail length observed during glu/gly stimulation. Gene labels were added for all points with a log_2_ fold-change in expression of >1.3 or <−1.0, or log_2_ fold-change in tail length of >0.3. (*F*) Comparison of change in expression with change in tail length observed during BDNF stimulation, otherwise as in *E*.

The primary visual cortex is composed of many cells of distinct lineages, including many nonneuronal cells, which can also respond to stimulation (Hrvatin et al. 2018). Thus, our conclusions regarding the tail-length changes observed in the visual cortex were not specific to neurons, and indeed were most likely an integration of many responses. To examine a population enriched for neurons, we measured the tail-length changes occurring in DIV14 cortical cultures 30 min after stimulation with either glu/gly or BDNF (Fig. 6). Inter-sample correlations of mean tail lengths improved in the culture experiment (Fig. 6A,B, *R*_S_ > 0.81) compared to the light-stimulation experiment (Supplemental Fig. S5C, *R*_S_ < 0.74), perhaps because each sample contained a mixture of cells from approximately five animals rather than tissue from a single animal, or perhaps because the culture and treatment methods were not prone to the same amount of variability as light stimulation.

Although levels of most mRNAs remained relatively constant at this 30-min timepoint, both treatments caused increased accumulation of some mRNAs, which tended to be mRNAs of annotated IEGs, suggesting that their increased accumulation was through transcriptional induction (Fig. 6C–F). These mRNAs with increased accumulation tended to have increased mean tail lengths, as expected for an influx of recently transcribed molecules. For slightly younger (DIV10) cortical cultures stimulated for 10 or 30 min with potassium chloride, results were similar, although few changes in tail length or abundance were observed at 10 min (Supplemental Fig. S7A–F). The magnitude of stimulus-dependent changes to tail length also decreased, perhaps because of the different stimulus or because less-mature cells were used. The greater tendency for increased tail lengths among mRNAs induced in culture compared to those induced in vivo was attributable to the earlier timepoint of the in vitro culture experiment, with the idea that after simulation for only 30 min these mRNAs were captured prior to their return to steady-state tail lengths. This explanation implies that many rapidly induced mRNAs can achieve steady-state tail lengths by 1 h but not by 30 min, although we cannot rule out the possibility that differences between the mouse and culture systems, such as distinct rates of mRNA decay, might underly these differences.

Some mRNAs underwent increased tail length without a substantial change in accumulation (e.g., *Pnrc1* after each 30-min stimulation), suggesting extension of their tails through cytoplasmic polyadenylation. Despite the three classes of mRNA tail-length behaviors observed in response to stimulation in both the tissue and primary-culture experiments, many of the mRNAs that underwent either increased tail length or increased accumulation (or both) did not do so consistently across the two types of experiments, which is attributable to differences in the identities and contexts of the cells as well as differences in the nature and strength of the signals. Comparisons of tail-length changes to TE changes did not provide evidence that tail length changes mediated detectable TE changes (Supplemental Fig. S7G–J, *R*_S_ ≤ 0.03).

### TE correlates with codon composition in neurons

**FIGURE 7.**
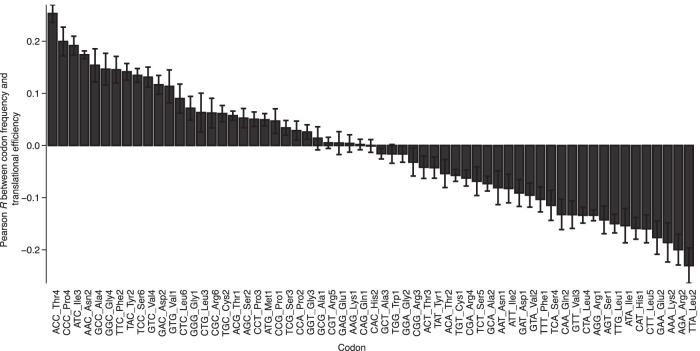
Codon composition explains some of the variation in TE observed in cortical cultures. The relationship between TE and codon frequency was determined for each of the 61 codons, using results from each of the six DIV14 primary-culture samples examined in this study. Plotted for each codon is the mean Pearson *R* value of the relationship (error bars, standard deviation), arranged in descending order.

In embryos and post-embryonic tissues, TE correlates with codon composition (Bazzini et al. 2016; Li et al. 2019), raising the question of whether TE and codon composition might also be related in neurons. When comparing the frequency of each codon in each mRNA with the TE of each mRNA, some codons were associated with better TE, and some with worse, with all six DIV14 primary culture samples showing similar trends (Fig. 7). The codons associated with better TE were primarily those that ended in either G or C, whereas the codons associated with worse TE were primarily their synonymous counterparts ending in either A or U. Part of the reason for this trend is that, as observed in other contexts (Burow et al. 2018; Fornasiero and Rizzoli 2019), codons ending in either G or C tend to co-occur with each other in mRNAs expressed in cortical culture neurons, as do codons ending in either A or U (Supplemental Fig. S8A). Notable exceptions to these trends were the UUG Leu codon, which co-occurs with A/U-ending codons despite ending in a G, and the AGG Arg codon, which is associated with poor TE despite ending in a G and does not co-occur with either the G/C-ending or the A/U-ending codons (Fig. 7; Supplemental Fig. S8A).

Despite this broad trend and the co-occurrence of codons with the G/C- or A/U-ending groups, some codons within these groups were much more predictive of strong or weak TE than others. One codon that correlated with poor translation was the AAA Lys codon, which is associated with ribosome “sliding” and reduced translation in *Escherichia coli* and *Saccharomyces cerevisiae* (Koutmou et al. 2015) and sometimes causes stalling (Kostova et al. 2017). In contrast, the other Lys codon, AAG, had no bias toward better or worse TE in our analysis and is reported to not be translated poorly (Koutmou et al. 2015). Three other codons associated with poor TE in our analysis, TTA, ATA, and CTA, are reported to be used relatively rarely in the transcriptome of HEK293T cells and generally excluded from the ribosomal-protein genes, which can be efficiently translated (Saikia et al. 2016).

The codon associated with the second-worst translation, the AGA Arg codon, was previously observed to induce ribosome pausing in the mouse strain used in our study (C57BL6J) due to a single-nucleotide polymorphism in the *n-Tr20* tRNA, an isodecoder for Arg (Ishimura et al. 2014). The observation that this codon was associated with poor TE in our data sets is notable, as ribosome pausing should be associated with increased RPF density. Apparently, correlation between this codon and poor TE was so strong that it overwhelmed any increased RPF density that stalling might have caused.

To explore further the possibility that pausing at more slowly translocating codons might increase ribosome density on mRNAs thereby inflating the apparent TE values of these mRNAs, we examined the relationship between pausing, as inferred from enrichment of the codon at the RPF position corresponding to the ribosomal A site (Tunney et al. 2018), and association of the codon with greater TE. For the cortical-tissue sample, the inferred translocation rates explained a substantial fraction of the variance in codon correlation with TE (*R*^2^ = 0.31), but for the cortical culture sample, they explained much less of the variance (*R*^2^ = 0.04). (Supplemental Fig. S8B,C).

Relationships between TE and amino acid composition were also observed (Supplemental Fig. S8D), although they were not as strong as those observed for codons, as expected when considering that the amino acid analysis cannot capture the variability observed between synonymous codons. Two of the three amino acids most correlated with poor TE were Lys and Arg, even though these amino acids make electrostatic interactions with the ribosome exit tunnel that slow elongation rate, which would presumably increase RPF density (Lu and Deutsch 2008). Furthermore, Glu was also associated with poor TE, despite its negative charge.

### A linear model predicts TE in neurons

The TE values that we observed in neurons and neuronal tissues, combined with our data sets measuring tail length, provided the opportunity to expand and evaluate the mRNA features associated with translation in neurons and compare them to nonneuronal tissues. In budding yeast, a study analyzing ribosome-profiling results found that 81% of the variation attributable to translational control could be explained by a linear combination of features describing CDS length, motifs in the 5′ UTR, codon composition, predicted folding energy, and uORF prevalence (Li et al. 2019). Another study in yeast showed that mRNA abundance also correlates with TE (Weinberg et al. 2016). In other eukaryotes, proline content, kozak and 5′ UTR-sequence composition, G:U wobble pairing in codons, CDS and UTR length, mRNA expression, poly(A)-tail length, RNA structure, and protein structure also correlate with TE (Reuveni et al. 2011; Qian et al. 2012; Shah et al. 2013; Stadler and Fire 2013; Artieri and Fraser 2014; Pop et al. 2014; Gritsenko et al. 2015; Li et al. 2019; Riba et al. 2019). However, models in mammals are not as explanatory as those in yeast—the best explaining 40%–46% of TE variation (40% in 3T3, 46% in mouse liver and kidney [Li et al. 2019]).

**FIGURE 8.**
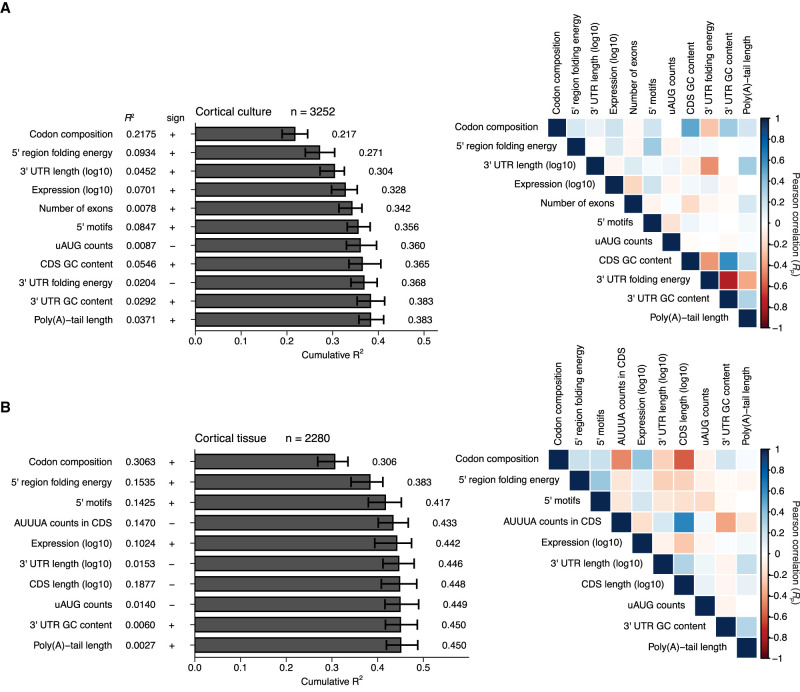
Linear models quantifying correlates of translation in cultured neurons and adult cortex. (*A*) Model for cultured primary cortical neurons. (*Left*) Sequence features used to predict TE in a primary cortical culture sample are arranged in the order in which they were selected during stepwise linear regression. Each feature is shown at the *left*, along with its *R*^2^ value in the absence of other features, the sign of its correlation with TE, and a bar plot of the cumulative *R*^2^ of a model built using the feature and more predictive features. Error bars indicate the 95% confidence interval on each feature obtained after bootstrapping the data 100 times. (*Right*) Pearson correlations for pairwise comparisons between each feature selected during the stepwise regression and all other selected features are arranged in a heatmap, with colors indicating the strength of each correlation (key). (*B*) Model for adult cortex; otherwise as in *A*.

**FIGURE 9.**
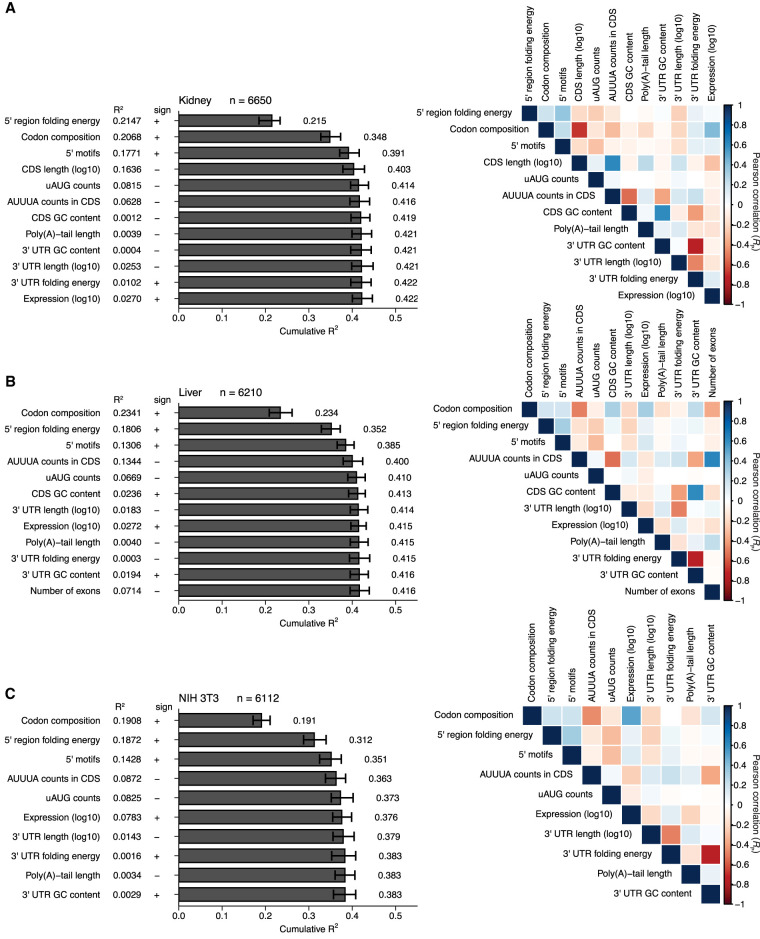
Linear models quantifying correlates of translation in nonneuronal systems. (*A*–*C*) Models for mouse kidney (Castelo-Szekely et al. 2017), mouse liver (Janich et al. 2015), and NIH 3T3 cells (Subtelny et al. 2014), respectively. Otherwise, these panels are as in Figure 8.

Less has been known about features that explain the TE variability observed among neuronal mRNAs. A study analyzing polysome-profiling results for alternative mRNA isoforms from neuronal cultures derived from human embryonic stem cells (hESCs) finds that CDS length, 3′-UTR length, and 5′ UTR length most consistently correlate with translation, with CDS length correlating positively with translation and UTR length correlating negatively (Blair et al. 2017). However, a model that considers these and other mRNA features to predict TE differences of neuronal mRNAs from different genes had not been developed. To fill this gap, we used multiple linear regression to build such a model, with the goal of providing a framework for considering the relative impact of factors contributing to translational control in neurons (Fig. 8). We focused on data from two sources: an unstimulated primary cortical culture and the adult cortical tissue, reasoning that these two samples represented the two distinct classes observed in our study (Fig. 2C). For comparison, we used published data sets (Subtelny et al. 2014; Janich et al. 2015; Castelo-Szekely et al. 2017) to build models from three nonneuronal tissues: liver, kidney, and NIH 3T3 cells (Fig. 9), for a total of five models.

Our models were constructed using two rounds of stepwise linear regression, using a procedure resembling that used to investigate correlates of mRNA stability (Spies et al. 2013). In the first round, informative features were sequentially selected from a menu of 17 possibilities based on the extent to which their inclusion improved the model. This stepwise inclusion of additional features continued until the selected feature no longer decreased the Akaike Information Criterion (AIC), which balances model complexity with predictive power (Akaike 1974). The AIC-selected lists of features were further filtered to retain only those for which inclusion added at least 0.003 to the overall *R*^2^ in at least one of the five models. Because of our interest in poly(A)-tail length, this feature was also retained, even though it did not pass this inclusion criterion. A second round of stepwise linear regression was then performed using this more restricted menu of 13 features to yield our final models (Figs. 8, 9). These final models each retained the 10–12 features most informative for its data set and had a cumulative *R*^2^ value within 0.004 of that for the corresponding model that considered all 17 of the original features (Supplemental Fig. S9). The final models each included 7–9 simple features as well as three complex features: codon composition, 5′ UTR motifs, and predicted folding energy of the 5′ UTR region (i.e., the 5′ UTR and the first 35 nt of the CDS [Li et al. 2019]). Each complex feature was composed of subfeatures; rather than incorporating the subfeatures individually, they were incorporated as a group, after training the model on the entire set of subfeatures. For example, rather than incorporating each codon abundance individually, a codon-composition model was trained on the 61 abundances of each codon in each coding sequence, and the coefficients of this model were used to build the full model. Each model was trained and evaluated 100 times—each time with different subsets of the data randomly assigned to training and test sets, and the mean *R*^2^ value from this bootstrapping is reported.

The most informative feature for predicting TE in both the cortical-culture and the adult-cortex samples was codon composition (Fig. 8A,B, *R*^2^ = 0.22 and 0.31, respectively). Its contribution was similar to that observed for models of translational efficiency in some other mouse cells and tissues (Li et al. 2019) (Fig. 9A–C, *R*^2^ = 0.19–0.23). The next most informative feature in both neuronal models was predicted folding energy of the 5′ UTR region (*R*^2^ = 0.09 and 0.15 for cortical culture and adult cortex, respectively). It positively correlated with TE, which indicated that a more structured 5′ UTR region leads to lower TE, consistent with previous observations (Pop et al. 2014; Weinberg et al. 2016; Li et al. 2019). In all models except for the kidney model, codon composition was more predictive than predicted folding energy.

In several respects, the adult-cortex model resembled the kidney, liver, and NIH 3T3 models more closely than it resembled the cortical-culture model. In cortex, kidney, liver, and 3T3 models, the motifs in the 5′ UTR were the third-most informative feature. In contrast, 3′-UTR length substantially contributed to the cortical-culture model (an *R*^2^ increase of 0.033 and its third-most informative feature) but added little to the other models (*R*^2^ increases ≤0.004).

When considered on its own, poly(A)-tail length correlated with TE in cortical culture substantially more than it did in cortex (Figs. 4, 8, *R*^2^ = 0.037 and 0.0027, respectively). However, at the step at which poly(A)-tail length was incorporated into each model, it did not contribute (Fig. 8). This diminution of the explanatory power for poly(A)-tail length in cortical culture was attributed to the correlation between poly(A)-tail length and more informative features, such as 3′-UTR length and folding energy, that had been incorporated earlier into the model. Thus, after accounting for these more informative features, poly(A)-tail length provided little additional explanatory power.

Considering all informative features together yielded models for cortical culture and adult cortex with median *R*^2^ values of 0.38 and 0.45, respectively (Fig. 8A,B), which resembled the values observed for other mouse samples (Fig. 9A–C, *R*^2^ = 0.42, 0.42, and 0.38 for kidney, liver, and 3T3 cells). These results suggested that the TEs of mRNAs in neuronal samples are not fundamentally less well understood (when considered in terms of our ability to predict them) than those of nonneuronal samples.

## DISCUSSION

One goal of this study was to build a predictive framework for translational control in mammalian neurons. As with analogous models developed for predicting TE in other mammalian cells and tissues, the *R*^2^ values of our models were <0.50, which indicated that most of the variance in translational regulation observed between mRNAs from different genes remained unexplained. In contrast, models developed for predicting TE in yeast explain as much as 81% of the variance (Li et al. 2019), despite the more limited range of TE in yeast. Some of the unexplained variation is attributable to experimental noise, but the reproducibility of our measurements, and the general agreement with similar studies from the literature, suggest that we and others are still missing (or are poorly parameterizing) major features of mRNAs that control TE in mammalian cells. One such feature might be mRNA structure. In our study, GC content and computationally predicted folding were used as a proxy for structural accessibility in the 5′ UTR region and the 3′-UTR, but more accurate accounting for structure and finer definition of the mRNA positions most sensitive to structure will presumably increase the power of the models.

Our study cannot speak to whether the features of our linear models are causative, rather than just correlative. For example, mRNA expression was selected as informative, even though this feature most likely did not directly influence TE, since the translation regulatory machinery presumably does not have a mechanism to detect whether or not an mRNA molecule is one of many from the same gene. Nonetheless, a correlation between mRNA expression and TE was observed, presumably because greater TE coevolved with higher mRNA expression to achieve high production of some proteins. Such evolutionary processes might even invert the observed relationship between a feature and TE. For example, AGA and AAA codons were associated with reduced TE, which suggested that any increased RPF density caused by slower decoding or stalled translation was more than offset by a noncausative correlation presumably established from evolutionary pressure to deplete these codons in ORFs that should be efficiently translated.

As observed in other contexts (Bazzini et al. 2016; Li et al. 2019), one of the more informative mRNA features in neurons was codon composition. The dominance of this feature seemed at odds with the view that TE differences stem largely from differences in translation initiation (Shah et al. 2013)—although because our models still explain less than half of the TE variance, we cannot exclude the possibility that missing features that explain the remaining variance might better parametrize initiation. To the extent that the correlation between codon composition and TE, with G/C ending codons associated with greater TE, cannot be explained by differential translocation rates, its cause is unknown. Perhaps mRNAs that must be expressed highest are under evolutionary pressure to have more structured ORFs.

Although neuronal mRNAs had diverse and reproducible TEs, stimulation altered TE of only a handful of mRNAs, and even these mRNAs had only modest TE changes. Instead, differential transcription explained most gene-expression changes observed in response to stimulation—at least when examining entire cells or bulk tissues after 30 min and 1 h, respectively. In contrast, a ∼60% reduction in ^35^S-methionine labeling is reported in isolated synaptosomes after 5 min of stimulation (Scheetz et al. 2000), which suggests that either profiling at earlier time points or profiling of isolated subcellular synaptic compartments might provide additional insight into stimulus-dependent changes in TE.

At face value, our observation of a correlation between tail length and TE in cultured neurons and neuronal tissues supported the notion that the length of the poly(A)-tail influences translation in neurons. Indeed, the lack of any positive correlation observed between tail length and TE in the mammalian samples previously profiled (Subtelny et al. 2014) implies that the relationship observed here might be unique among tissues of the adult mouse. However, this relationship, although statistically significant, was modest, explaining less than 5% of the variance in TE for the most correlated samples. Moreover, after accounting for the correlation between tail length and other features more informative for predicting TE, tail length explained almost none of the remaining variance, which raised additional questions regarding the prospect that poly(A)-tail length influences TE in neurons.

Despite the lack of a strong relationship between TE and poly(A)-tail length, several mRNAs, such as *Spred1* or *Pncr1*, did appear to undergo cytoplasmic polyadenylation in response to stimulation. One difference between our results and those reported previously (Wu et al. 1998; Du and Richter 2005; Dziembowska et al. 2012; Udagawa et al. 2012) is that we did not observe a significant change in the tail length of *Camk2a* in response to stimulation, either in neuronal culture or in brain tissues. This difference might be attributed to a difference in our stimulation protocols, or a difference between mice and rats, used in our study and previous studies, respectively.

Another difference between our results and those of others that report cytoplasmic polyadenylation in the brain (Wu et al. 1998; Du and Richter 2005; Dziembowska et al. 2012; Udagawa et al. 2012), is that we did not find evidence for increased TE for those mRNAs with lengthened tails. Nonetheless, as mentioned earlier with respect to TE differences more generally, we cannot exclude the possibility of localized coupling between tail length and TE. For example, although cytoplasmic polyadenylation might extend the tails of most mRNAs from a particular gene, perhaps only transcripts that are in the dendrites produce more protein as a result of their longer tails. Supporting this idea, cytoplasmic polyadenylation is reportedly detected in both the rat visual cortex and cultured hippocampal neurons without additional subcellular purification (Wu et al. 1998; Du and Richter 2005), but corresponding increases in protein abundance are typically reported only in synaptosomes (Wu et al. 1998; Dziembowska et al. 2012). Indeed, the initial studies that proposed a relationship between tail length and TE for *Camk2a* and a handful of other mRNAs did so while arguing for a dendrite-specific role for this phenomenon, with the idea that cytoplasmic polyadenylation, working together with tail-length-mediated control of translation, could tune translational output on a timescale more rapid than that required for mRNA transcription, processing, and transport to the dendrites (Wu et al. 1998; Du and Richter 2005; Dziembowska et al. 2012; Udagawa et al. 2012). Perhaps PABPC, the mRNA-decay machinery, and the translation machinery are less abundant in the distal regions of the neuron, thereby creating the conditions shown to be required for tail length to influence TE in other contexts (Xiang and Bartel 2021). Supporting this idea, calculations based on dendritic volume suggest that concentrations of most macromolecules differ between the dendrite and soma (Kosik 2016). Experimental assessment of localized tail-length control of TE currently faces technical challenges. For example, synaptosome or synaptoneurosome preparations are complex and variable (Dunkley et al. 1988; Chicurel et al. 1993; Bagni et al. 2000; Villasana et al. 2006; Westmark et al. 2011), with 58%–70% of these preparations consisting of resealed, vesicle-loaded synaptosomes or isolated nerve terminals, as determined by electron microscopy (Nagy and Delgado-Escueta 1984; Wilhelm et al. 2014). However, once suitable biochemical or imaging methods have been developed, the dendrite would be a promising place to look for localized tail-length control of TE.

## MATERIALS AND METHODS

### Cell lines and cell culture for transformed lines

HEK293FT cells (Thermo Fisher) were used for packaging lentivirus. These cells were grown at 37°C in 5% CO_2_ in DMEM supplemented with 10% FBS (Takara). HEK293 cells are female with a complex karyotype. No mycoplasma testing was performed.

### Methods details

#### Mouse husbandry

Mice for primary culture or tissue samples were group-housed (fewer than five mice per cage, cohousing females or sibling males) in a 12 h light–dark cycle (light between 07:00 and 19:00) in a temperature-controlled room (21.1 ± 1.1°C) at the Whitehead Institute for Biomedical Research with food and water ad libitum. Mice were maintained according to protocols approved by the Massachusetts Institute of Technology Committee on Animal Care. Euthanasia was performed by CO_2_ inhalation except for neonates, where rapid decapitation was used. Sex was not determined for embryos or neonatal pups. For analyses of older animals (non-neonatal), only male mice were used. Housing and maintenance of mice for light-stimulation experiments was performed as previously described (Hrvatin et al. 2018).

#### Primary cortical culture

Primary neurons were cultured as described (Beaudoin et al. 2012) with slight modifications. The six samples used in this study were prepared in two batches of three samples from two litters per batch. Cortices were dissected from male and female P0–P1 mouse pups in ice-cold dissection medium (Hanks Balanced Salt Solution, Thermo Fisher, supplemented with 1 mM sodium pyruvate, 0.1% glucose, and 10 mM HEPES, pH 7.3) and washed twice with ice-cold dissection medium. Samples were trypsinized by incubating half of the cortices in each of two 15 mL conical tubes containing 5 mL dissection medium and trypsin (0.25% w/v final, Worthington) for 20 min at 37°C. DNase I (0.1% w/v final, Sigma-Aldrich) was added, and the sample was incubated for 2–5 min to prevent clumping. Tissue was washed again in room-temperature dissection medium at which point the medium was exchanged with 10 mL of plating medium (Basal Medium Eagle, Thermo Fisher, supplemented with 20% FBS, 0.45% glucose, 1 mM sodium pyruvate, 2 mM GlutaMAX, Thermo Fisher, and 100 U/mL penicillin/streptomycin), taking care not to allow the tissue to become exposed to air. The tissue was then triturated in two 15 mL conical tubes with an FBS-coated 5 mL serological pipet in three rounds, performing no more than 10 triturations per round and transferring the dissociated cells to a new 15 mL conical tube between each round. Dissociated cells were passed through a 70 µm cell strainer, counted, and plated in plating medium at ∼100,000 cells/cm^2^ on 15 cm tissue-culture plates coated with poly-D-lysine (50 µg/mL). Cells were cultured only if viability, assessed by Trypan Blue staining, exceeded 80% upon plating. Plating medium was exchanged for maintenance medium (Neurobasal, Thermo Fisher, supplemented with 1X B27 Serum-free supplement, and 2 mM GlutaMAX), between 2–6 h after plating. Two days after plating, cells were treated with 5 µM AraC for 24 h, and then maintenance medium was fully exchanged. Neurons were fed using half-media exchanges every 3 d thereafter. All neurons in this study were harvested at DIV14 (with the day of plating considered DIV1), except for those used in the KCl-stimulation experiments, which were harvested at DIV10.

#### Lentiviral production and transduction

Vectors expressing miRNAs from the 3′-UTR of *EGFP* were cloned as follows. The sequences of pre-miR-1a-1 or pre-miR-155 along with 100 bp of flanking genomic sequence were amplified from mouse genomic DNA with primers adding EcoRI restriction sites (Supplemental Table S1). These segments were cloned immediately downstream from the *EGFP* coding sequence in a lentiviral plasmid expressing *EGFP* under the control of a synapsin promoter (originally derived from addgene #60955, replacing the promoter and insert casette with the synapsin–*EGFP* cassette from addgene #58867 using restriction-enzyme cloning).

The day prior to transfection, 293FT cells were plated onto 15 cm dishes at a density of 18,000 cells/cm^2^. For transfection, each mix contained 9 µg shuttle vector, 9 µg of pCMV-dR8.91 packaging plasmid (a gift from Jonathan Weissman), and 1 µg envelope plasmid pMD2.G (a gift from Didier Trono; Addgene #12259) in 1 mL of Optimem (Thermo Fisher) and 125 µL of FuGene HD (Promega). After incubation with transfection mix for 15 min at room temperature, maintenance-medium was removed from cells and they were re-fed with 20 mL of Optimem containing 10% FBS. The following day, the Optimem mixture was removed and cells were re-fed with 30 mL of DMEM containing 10% FBS and grown for two more days, at which point the medium was collected and cleared of cell debris by filtering through a 0.45 µM PES syringe filter, using two filters per supernatant to prevent clogging. The ∼30 mL of viral supernatant was concentrated by addition of 10 mL of Lenti-X Concentrator (Takara), overnight incubation at 4°C, and centrifugation at 1500*g* for 45 min in a swinging-bucket rotor in a 50 mL conical tube. After discarding the supernatant, the pellet was resuspended in 1 mL of ice-cold PBS, aliquoted into 100 µL aliquots, flash frozen, and stored at −80°C. Neurons were transduced by infecting with 800 µL of virus at DIV5 (80% of the total virus) in a 15 cm plate.

#### Small-RNA blots

Small-RNA blots were performed as described (Kleaveland et al. 2018). Briefly, total RNA (1 µg) from miRNA-transduced neuronal cultures was denatured with 2X formamide loading dye (Thermo Fisher) and resolved on a 15% urea-polyacrylamide gel. RNA was then transferred onto an Amersham Hybond-NX neutral nylon membrane (GE Healthcare) using a semi-dry transfer apparatus (BioRad). Membranes were crosslinked with 0.466 g EDC (N-(3-dimethylaminopropyl)-N0-ethylcarbodiimide; Thermo Fisher) dissolved in 15 mL of 125 mM 1-methylimidazole for 1 h at 60°C and then preincubated with ULTRAhyb-Oligo Buffer (Thermo Fisher) for 30 min at 42°C with rotation. 5′ radiolabeled probe (a DNA oligo complementary to the miRNA sequence) was then added, and the membrane was hybridized overnight with rotation at 42°C. The next morning, membranes were rinsed twice with low-stringency buffer (2× SSC and 0.1% SDS), then incubated for 30 min under rotation at 42°C, rinsed twice with high-stringency buffer (0.1× SSC and 0.1% SDS), and incubated for 30 min under rotation at 42°C. The blots were then exposed to a phosphorimaging screen for several hours or overnight. Signal was detected using the Typhoon FLA 7000 phosphorimager (GE Healthcare Life Sciences). Membranes were stripped before hybridizing to another probe. To do so, they were incubated four times with 100 mL of boiling stripping buffer (0.1% SDS, 0.1× SSC) for 10 min each time with vigorous shaking. Membranes were rinsed with water to remove residual SDS, exposed to a phosphorimager screen to check the residual signal, and then probed as above.

#### Linear model

Multiple linear regression was performed similarly to Spies et al. (2013). Prior to modeling, variables were log transformed if indicated. The predicted RNA folding energy and 5′ motif features for the mouse transcriptome were generated as described and not regenerated for this study (Li et al. 2019). 3′-UTR lengths were determined from PAL-seq annotations using the eight light-stimulation visual cortex PAL-seq data sets. Predicted 3′-UTR folding energy was calculated using the Vienna package (Lorenz et al. 2011), normalizing the minimum free-energy values by UTR length. For mRNAs with multiple 3′-UTR isoforms, 3′-UTR length and folding energy were calculated by averaging all mRNAs from one gene, weighted by expression.

After compiling the values for TE and each feature for a particular sample, the Akaike Information Criterion (AIC) was used to order the features and to remove those that did not contribute significantly. This was done using the stepAIC algorithm from the MASS package in R. For the final model figures, features were removed if they increased the resulting adjusted *R*^2^ (adjusted for number of features using the ‘lm’ command in R) by less than 0.003, even if they met the AIC threshold for inclusion. When building models from all features regardless of their contribution (Supplemental Fig. S9), features were added according to the magnitude of the adjusted *R*^2^ value obtained for that feature and TE, starting with the strongest correlating feature. Confidence intervals were determined by randomly selecting a subset of genes and recalculating the correlation 100 times using the *boot* package in R (Davison and Hinkley 1997). The correlation heatmaps (Figs. 8, 9, right) were prepared using the *corrplot* package in R (Wei and Simko 2017).

#### Mouse visual cortex

Total RNA from microdissected visual cortex samples also used to prepare scRNA-seq libraries for a separate study (Hrvatin et al. 2018) were a kind gift from Sinisa Hrvatin and Michael Greenberg. For each sample, total RNA was isolated using the RNeasy Mini (Qiagen) procedure according to the manufacturer's instructions.

#### Tissue preparation

Hippocampi from E16 embryos were dissected individually into Eppendorf tubes and flash frozen. The adult hippocampus from a male mouse was dissected and flash frozen. Adult cortex from a male mouse was flash frozen. Samples were stored at −80°C until lysate preparation.

To prepare lysate, frozen tissue was transferred to a 2 mL Dounce homogenizer filled with ribosome-profiling lysis buffer (10 mM Tris-HCl, pH 7.4, 5 mM MgCl_2_, 100 mM KCl, 1% Triton-X 100, 2 mM DTT, 100 µg/mL cycloheximide, 500 U/mL RNasein Plus [Promega], and cOmplete Mini EDTA-free Protease Inhibitor Tablets [Roche, 1 tablet/10 mL]) (Subtelny et al. 2014). Samples were then homogenized with 10 strokes of pestle A followed by 10 strokes of pestle B, taking care not to introduce bubbles into the buffer. Following homogenization, the sample was transferred to two Eppendorf tubes, centrifuged at 1300*g* for 10 min, and the supernatant was aliquoted and flash frozen at −80°C for use in ribosome profiling, RNA sequencing, and tail sequencing. For RNA and tail sequencing, RNA was extracted by adding five volumes of TRIzol (Thermo Fisher) to the frozen lysate, allowing it to thaw to room temperature, and continuing the preparation according to the manufacturer's instructions.

#### Tail-length measurements

This study measured poly(A)-tail lengths from 20 samples: four light stimulated visual-cortex samples and four controls, two miRNA-transduced primary cortical-culture samples and a GFP-transduced control, a BDNF-stimulated cortical-culture sample, a glu/gly-stimulated cortical-culture sample and an associated unstimulated control sample, two KCl-stimulated samples at different time points and an associated unstimulated sample, and one sample each from adult hippocampus, adult cortex, and embryonic hippocampus. PAL-seq v2 (Eisen et al. 2020b) was used for the eight visual-cortex samples and the adult and embryonic hippocampal samples. TAIL-seq (Chang et al. 2014) was used for the nine primary cortical-culture samples and the adult-cortex sample. The two techniques were used because they were being sequentially developed while this work was in progress. Because both techniques rely on Illumina base calling to determine the tail length, they have similar sensitivities.

#### PAL-seq v2

Library preparation was as described previously (Eisen et al. 2020b) and summarized as follows. An amount of 20–30 μg of total RNA was used to prepare PAL-seq libraries. Tail-length standard mixes (1 ng of set 1 and 2 ng of set 2), and trace 5′-radiolabeled marker RNAs (Supplemental Table S1) were added to each sample to assess tail-length measurements and ligation outcomes, respectively. Polyadenylated ends, including those with a terminal uridine, were ligated to a 3′-biotinylated adaptor DNA oligonucleotide (1.8 μM) in the presence of two splint DNA oligonucleotides to capture tails ending in either A or U (1.25 and 0.25 μM for the two respective splint oligonucleotides, Supplemental Table S1) using T4 Rnl2 (NEB) in an overnight reaction at 18°C. Following 3′-adaptor ligation, the RNA was extracted with phenol–chloroform (pH 8.0), precipitated, resuspended in RNase T1 sequence buffer (Thermo Fisher), heated to 50°C for 5 min and then put on ice. RNase T1 was then added to a final concentration of 0.006 U/μL, and the reaction was incubated at room temperature for 30 min, followed by phenol–chloroform extraction and RNA precipitation. Precipitated RNA was captured on streptavidin beads, 5′ phosphorylated, and ligated to a 5′ adaptor as described (Subtelny et al. 2014), but using a modified 5′ adaptor sequence (Supplemental Table S1). Following reverse transcription using SuperScript III (Invitrogen) with a barcode-containing DNA primer, cDNA was purified using PAGE as described (Subtelny et al. 2014), selecting a 160–810 nt size range. Libraries were amplified by PCR using Titanium Taq polymerase (Takara) according to the manufacturer's protocol with a 1.5 min combined annealing/extension step at 57°C. PCR-amplified libraries were purified using AMPure beads (Agencourt, 40 μL beads per 50 μL PCR, two rounds of purification), according to the manufacturer's instructions.

PAL-seq v2 libraries were sequenced on an Illumina HiSeq 2500 operating in rapid mode. Hybridization mixes were prepared with 0.375 fmol PCR-amplified library that had been denatured with standard NaOH treatment and brought to a final volume of 125 μL with HT1 hybridization buffer (Illumina, 3 pM library in final mix). Clusters were generated and the sequencing primer was hybridized according to the standard protocol. For the splinted-ligation libraries, two dark cycles were performed (i.e., two rounds of standard sequencing-by-synthesis in which imaging was skipped), which extended the sequencing primer by 2 nt, thereby enabling measurement of poly(A) tails terminating in nonadenosine bases.

Following the dark cycles, a custom primer-extension reaction was performed on the sequencer using 50 μM dTTP as the only nucleoside triphosphate in the reaction. To perform this extension, the flow cell temperature was first set to 20°C. Then, 120 μL of universal sequencing buffer (USB, Illumina) was flowed over each lane, followed by 150 μL of Klenow buffer (NEB buffer 2 supplemented with 0.02% Tween-20). Reaction mix (Klenow buffer, 50 μM dTTP, and 0.1 U/μL Large Klenow Fragment, NEB) was then flowed on in two aliquots (150 and 100 μL). The flow-cell temperature was then increased to 37°C at a rate of 8.5°C per min and the incubation continued another 2 min after reaching 37°C. A total of 150 μL of fresh reaction mix was then flowed in, and following a 2 min incubation, 75 μL of reaction mix was flowed in eight times, with each flow followed by a 2 min incubation. The reaction was stopped by decreasing the flow cell temperature to 20°C, flowing in 150 μL of quench buffer (Illumina HT2 buffer supplemented with 10 mM EDTA) and then washing with 75 μL of HT2 buffer. The flow cell was prepared for subsequent sequencing with a 150 μL and a 75 μL flow of HT1 buffer (Illumina). Fifty cycles of standard sequencing-by-synthesis were then performed to yield the first sequencing read (read 1). XML files to configure a HiSeq 2500 for this protocol are provided at https://github.com/kslin/PAL-seq.

The flow cell was stripped, a barcode sequencing primer was annealed, and seven cycles of standard sequencing-by-synthesis were performed to read the barcode. The flow cell was then stripped again, and the same primer as used for read 1 was hybridized and used to prime 250 cycles of standard sequencing-by-synthesis to generate read 2. Thus, each PAL-seq tag consisted of three reads: read 1, read 2, and the indexing (barcode) read. For cases in which a tag corresponded to a polyadenylated mRNA, read 1 was the reverse complement of the 3′ end of the 3′-UTR and was used to identify the mRNA and cleavage-and-polyadenylation site of long-tailed mRNAs. The indexing read was used to identify the sample, and read 2 was used to measure poly(A)-tail length and identify the mRNA and cleavage-and-polyadenylation site of short-tailed mRNAs. The intensity files of reads 1 and 2 were used for poly(A)-tail length determination, along with the Illumina fastq files.

#### TAIL-seq

The library preparation for this protocol was the same as for PAL-seq v2, but the resulting cDNA was amplified using different primers (Supplemental Table S1) and sequenced using a different protocol. The first read of TAIL-seq involved sequencing the 3′-UTR from the gene body toward the tail, with the sequencing primer annealing to sequences added with the 5′ adaptor. This 5′ adaptor was an equimolar mixture of four sequences with different numbers of nucleotides between the primer-binding site and the insert (Supplemental Table S1) to ensure that highly abundant sequences (such as rRNA fragments) did not cause a large portion of the flow cell to fluoresce in a single channel. Amplification and purification were as for PAL-seq v2. Samples were sequenced with a paired-end 50-by-250 run using a HiSeq 2500 operating in normal mode using a v3 kit. Analysis was as described for PAL-seq v2, except a five-state GHMM was used (Chang et al. 2014) to accommodate the difference in the nature of the T-signal output imparted by the different mode of sequencing. The five states were an initiation state, a poly(A) state, a poly(A)-transition state, a non-poly(A) transition state, and a non-poly(A) state.

#### PAL-seq v2 data analysis

Tail lengths for the splinted-ligation data were determined using a Gaussian hidden Markov model (GHMM) from the python2.7 package ghmm (http://ghmm.org/), analogous to the model used in TAIL-seq (Chang et al. 2014) and described in the next paragraph. Read 1 was mapped using STAR (v2.5.4b) run with the parameters “–alignIntronMax 1 –outFilterMultimapNmax 1 –outFilterMismatchNoverLmax 0.04 –outFilterIntronMotifs RemoveNoncanonicalUnannotated –outSJfilterReads,” aligning to an index of the mouse genome built using mm10 transcript annotations that had been filtered to single representatives of each gene, selecting the longest transcript and removing all overlapping transcripts on the same strand (Eichhorn et al. 2014). The genome index also included sequences of the quantification spikes and the common portion of the poly(A)-tail length standards. The sequences that identified each RNA standard (the last 20 nt of each standard sequence, Supplemental Table S1) were not aligned using STAR. Instead, the unix program grep (v2.16) was used to determine which reads matched each standard (allowing no mismatches), and these reads were added to the aligned reads from the STAR output. Tags corresponding to annotated 3′-UTRs of mRNAs were identified using bedtools (v2.26.0), and if the poly(A)-tail read (read 2) contained a stretch of ≥10 T residues (the reverse complement of the tail) in an 11-nt window within the first 30 nt, this read was carried forward for GHMM analysis. If read 2 failed to satisfy this criterion but began with ≥4 T residues, the tail length was called based on the number of contiguous T residues at the start of read 2; by definition, these tails were <10 nt and thus easily determined by direct sequencing.

For each read 2 that was to be input into the GHMM, a “T signal” was first calculated by normalizing the intensity of each channel for each cycle to the average intensity of that channel when reading that base in read 1 and then dividing the thymidine channel by the sum of the other three channels. Sometimes a position in a read would have a value of 0 for all four channels. A read was discarded if it contained more than five such positions. Otherwise, the values for these positions were imputed using the mean of the five nonzero signal values upstream and downstream (10 positions total) of the zero-valued position. A three-state GHMM was then used to decode the sequence of states that occurred in read 2. It consisted of an initiation state (state 1), a poly(A)-tail state (state 2), and a non-poly(A)-tail state (state 3). All reads start in state 1. From state 1 the model can remain in state 1 or transition to state 2. From state 2 the model can either remain in state 2 or transition to state 3. The model was initialized with the transition probabilities shown in Table 1.

**TABLE 1.**
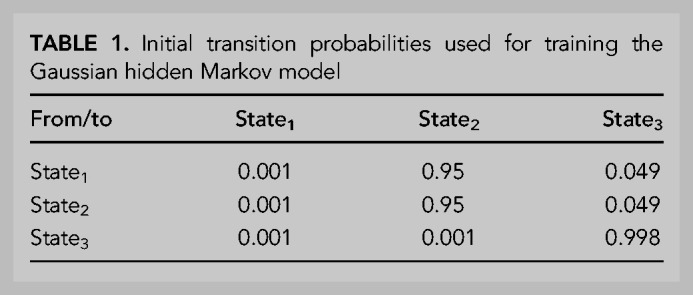
Initial transition probabilities used for training the Gaussian hidden Markov model

The initial emissions were Gaussian distributions with means of 100, 1, and −1 and variances of 1, 0.25, and 0.25, respectively. The emission Gaussians for the model corresponded to the logarithm of the calculated T signal at each sequenced base in read 2. The initial state probabilities were 0.998, 0.001, and 0.001 for states 1, 2, and 3, respectively. After initializing the model, unsupervised training was performed on 10,000 randomly selected PAL-seq tags, and then the trained model was used to decode all tags, with the number of state 2 cycles reporting the poly(A)-tail length for a tag. Only genes with ≥50 tail-length measurements were considered for analyses involving mean poly(A)-tail lengths. Documentation and code to calculate and analyze T signals and determine tail lengths are available for both the TAIL-seq and PAL-seq pipelines at https://github.com/kslin/PAL-seq.

Labeling of mRNAs (Fig. 6E,F; Supplemental Fig. S7E–F) was performed using the ggrepel package (Slowikowski 2020). Mapping reads to gene features (Fig. S1A) was performed using the RSeQC analysis software (Wang et al. 2012).

#### Annotation of 3′ ends

3′-end annotations were generated from PAL-seq tags from the eight visual cortex data sets. All data from these samples were combined, and mRNAs with tails ≥11 nt were used for annotation, using an algorithm previously developed for data from poly(A)-position profiling by sequencing (3P-seq) (Jan et al. 2011). Each PAL-seq read 1 that mapped (with at least 1 nt of overlap) to an annotated 3′-UTR (Eichhorn et al. 2014) was compiled by the genomic coordinate of its 3′-UTR nucleotide closest to the tail. The genomic coordinate with the most mapped reads was annotated as a 3′ end. All reads within 10 nt of this end (a 21-nt window) were assigned to this end and removed from subsequent consideration. This process was repeated until there were no remaining 3′-UTR-mapped reads. For each gene, the 3′-end annotations were used in subsequent analyses if they accounted for ≥10% of the 3′-UTR-mapping reads for that gene.

#### RNA-seq and ribosome profiling

Total RNA was either poly(A)-selected (adult and embryonic hippocampus samples) or Ribozero (Illumina)-depleted (six primary cortical-culture samples and the adult cortex sample). For ribosome-profiling libraries, 300–600 μL aliquots of lysate were digested with 0.3 U/μL RNase I (Ambion) for 30 min at room temperature and then run on a 10%–50% sucrose gradient to purify monosomes (Subtelny et al. 2014). RNAs from both RNA-seq and ribosome profiling were then size-selected, ligated to adapters, reverse-transcribed, and amplified (Subtelny et al. 2014). These libraries were sequenced on an Illumina HiSeq 2500. For all RNA-seq and ribosome profiling data, only reads mapping to ORFs of annotated gene models (Eichhorn et al. 2014) were considered, excluding the first 50 nt of each ORF. A cutoff of ≥10 reads per million mapped reads (RPM) was applied to each gene in each RNA-seq sample.

#### Calculation of miRNA-mediated repression

Secondary effects of expressing a miRNA can have a greater impact on mRNAs with longer 3′-UTRs relative to those with shorter 3′-UTRs (Agarwal et al. 2015), presumably because longer 3′-UTRs tend to contain more sites to other regulatory factors, including other miRNAs. As a result, 3′-UTR length differences can complicate the measurement of the repressive effects of an expressed miRNA. For this reason, we first normalized the fold-changes of all mRNAs based on their 3′-UTR length as in Eisen et al. (2020a). For all mRNAs without a 6-nt seed-matched site to the induced miRNA in the entire transcript (no-site mRNAs), the relationship between fold-change and the log of the 3′-UTR length was calculated using linear regression, and then the fold-changes of all mRNAs (with and without a target site) were normalized by their 3′-UTR lengths such that the slope of the relationship between no-site mRNAs and 3′-UTR length was 0. Normalized fold-changes for mRNAs containing at least one predicted miRNA target site in their 3′-UTR were then compared to those for the no-site mRNAs. For all mRNAs passing our expression threshold in the GFP-overexpression sample, we calculated the log_2_ fold-changes in mRNA abundance, RPF abundance, or poly(A)-tail length in samples from neurons transduced with either miR-155 or miR-1 compared to neurons transduced with GFP. The repressive effect of the miRNA on a set of predicted miRNA targets was then calculated by subtracting the median-normalized fold-change for no-site mRNAs from the mean-normalized fold-change for the set of predicted targets. Top targets were defined using RPF measurements from a previous study (Eichhorn et al. 2014), choosing from among the predicted targets those with expression that decreased to ≤75% of their original expression after 12 h of miRNA induction.

## DATA DEPOSITION

Raw and processed RNA-seq, ribosome profiling, PAL-seq, and TAIL-seq read data is available at the GEO, accession number GSE194172. Code for configuring an Illumina HiSeq 2500 machine for PAL-seq and for calculation of tail lengths from PAL-seq or TAIL-seq data is available at https://github.com/kslin/PAL-seq. Code for fitting models of TE is available at https://github.com/timeisen/TranslationInNeurons.

## SUPPLEMENTAL MATERIAL

Supplemental material is available for this article.

## Figures and Tables

**Timothy J. Eisen:**
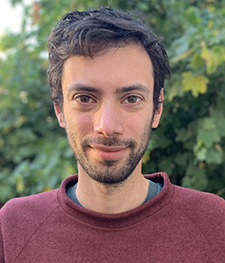


## MEET THE FIRST AUTHOR

***Meet the First Author(s)* is a new editorial feature within *RNA*, in which the first author(s) of research-based papers in each issue have the opportunity to introduce themselves and their work to readers of *RNA* and the RNA research community. Timothy J. Eisen is the first author of this paper, “The interplay between translational efficiency, poly(A) tails, microRNAs, and neuronal activation.” This work was conducted at the Whitehead Institute at MIT while Tim was a graduate student in Professor David Bartel's laboratory. Tim is now a postdoctoral researcher at UC Berkeley.**

**What are the major results described in your paper and how do they impact this branch of the field?**

Our paper investigates features underlying translational regulation in neurons. When examining neurons in bulk, we find that mRNA poly(A)-tail length correlates with translational efficiency (TE). However, this correlation is modest, and those mRNAs for which tail length regulates TE appear to be the exception rather than the rule. In the context of other mRNA features, such as codon composition, tail length has a negligible effect on TE when incorporated into a linear model. Additional experiments involving the mechanism of microRNA-mediated repression show that microRNAs destabilize neuronal transcripts, in keeping with cellular contexts in which the poly(A)-tail mediates mRNA decay, not TE. These results encourage future studies to examine additional correlates of TE in neurons, and search for neuronal contexts in which tail length has a major impact on TE.

**What led you to study RNA or this aspect of RNA science?**

When I joined Professor Bartel's lab as a new graduate student, the lab had just published an exciting study that showed that poly(A)-tail length is the major regulator of TE in oocytes and early embryos, but this regulatory mechanism is absent from the mature cell types and tissues examined (Subtelny et al. 2014). The study didn't look in neurons, however, and previous studies had implicated the poly(A) tail in regulating protein output in neurons in response to stimulation. I was very interested in learning and memory in the mammalian brain and decided to examine the role of the poly(A) tail in neurons using the recently developed tail-length sequencing techniques in the lab.

**During the course of these experiments, were there any surprising results or particular difficulties that altered your thinking and subsequent focus?**

For me, the most surprising finding of this work was the low correlation between tail-length and TE that we consistently observed in neuronal cultures. I found it surprising for two reasons. First, that we can observe the correlation at all puts the neuronal system in a unique place among the other mature tissues examined, none of which have any detectable relationship between tail length and TE. And second, because the correlation is so low, neurons do not resemble the oocyte or embryonic systems either. Hopefully, future studies examining subcellular compartments, different neuronal cohorts, or other times during neuronal activation will lead to a better understanding of tail-TE coupling.

**What are some of the landmark moments that provoked your interest in science or your development as a scientist?**

I remember when I was 11 years old and saw the journal *Nature*—the issue on the human genome project, with a cover image of DNA made up of small squares of humans—I decided that I wanted to be a geneticist. At the time, I didn't understand that much about genes, and I certainly didn't know much about the job of a geneticist, but I appreciated how groundbreaking understanding our genes could be. And I really wanted to be a part of the future efforts to gain this understanding.
